# Supplementary material for: Multi‐dataset identification of innovative feature genes and molecular mechanisms in keratoconus
Source: J Cell Mol Med. 2024 Sep 19;28(18):e70079. doi: 10.1111/jcmm.70079 (PMC11412914; doi:10.1111/jcmm.70079)
Supplement: Supplementary file 2 — Material S2. [file JCMM-28-e70079-s002.zip › 7.GSEA analysis/2.ATOH7/KEGG_ECM_RECEPTOR_INTERACTION.html]

Details for gene set KEGG\_ECM\_RECEPTOR\_INTERACTION[GSEA]

|  || Dataset | ATOH7.group.cls#h\_versus\_l |
| Phenotype | group.cls#h\_versus\_l |
| Upregulated in class | l |
| GeneSet | KEGG\_ECM\_RECEPTOR\_INTERACTION |
| Enrichment Score (ES) | -0.2028046 |
| Normalized Enrichment Score (NES) | -0.64849985 |
| Nominal p-value | 0.92479676 |
| FDR q-value | 1.0 |
| FWER p-Value | 1.0 |
Table: GSEA Results Summary

  

Fig 1: Enrichment plot: KEGG\_ECM\_RECEPTOR\_INTERACTION      
 Profile of the Running ES Score & Positions of GeneSet Members on the Rank Ordered List

  

| SYMBOL | TITLE | RANK IN GENE LIST | RANK METRIC SCORE | RUNNING ES | CORE ENRICHMENT || 1 | ITGA5 | na | 1306 | 0.407 | -0.0011 | No |
| 2 | COL4A6 | na | 2104 | 0.337 | 0.0061 | No |
| 3 | LAMA1 | na | 2673 | 0.300 | 0.0167 | No |
| 4 | ITGB8 | na | 3379 | 0.261 | 0.0198 | No |
| 5 | COL5A2 | na | 4272 | 0.227 | 0.0144 | No |
| 6 | ITGA1 | na | 4519 | 0.218 | 0.0268 | No |
| 7 | HMMR | na | 4920 | 0.204 | 0.0336 | No |
| 8 | ITGB3 | na | 5329 | 0.191 | 0.0390 | No |
| 9 | LAMB3 | na | 5412 | 0.188 | 0.0535 | No |
| 10 | COL4A4 | na | 5676 | 0.180 | 0.0621 | No |
| 11 | CD36 | na | 6081 | 0.169 | 0.0656 | No |
| 12 | COL5A1 | na | 6103 | 0.168 | 0.0801 | No |
| 13 | SV2C | na | 6470 | 0.158 | 0.0837 | No |
| 14 | CD44 | na | 6491 | 0.157 | 0.0972 | No |
| 15 | COL6A2 | na | 6518 | 0.156 | 0.1106 | No |
| 16 | CHAD | na | 6647 | 0.153 | 0.1206 | No |
| 17 | ITGA6 | na | 6683 | 0.152 | 0.1332 | No |
| 18 | LAMA3 | na | 7593 | 0.128 | 0.1185 | No |
| 19 | SDC3 | na | 8074 | 0.118 | 0.1152 | No |
| 20 | ITGA2 | na | 8085 | 0.117 | 0.1255 | No |
| 21 | CD47 | na | 8465 | 0.109 | 0.1243 | No |
| 22 | LAMC1 | na | 8509 | 0.108 | 0.1328 | No |
| 23 | COL11A1 | na | 8735 | 0.104 | 0.1356 | No |
| 24 | ITGA7 | na | 8847 | 0.102 | 0.1415 | No |
| 25 | ITGAV | na | 8959 | 0.100 | 0.1473 | No |
| 26 | LAMC2 | na | 9360 | 0.093 | 0.1441 | No |
| 27 | COL5A3 | na | 9458 | 0.091 | 0.1494 | No |
| 28 | ITGB1 | na | 9539 | 0.090 | 0.1552 | No |
| 29 | ITGB5 | na | 10348 | 0.076 | 0.1387 | No |
| 30 | ITGA3 | na | 10756 | 0.070 | 0.1332 | No |
| 31 | COL4A2 | na | 12318 | 0.048 | 0.0925 | No |
| 32 | LAMB1 | na | 12334 | 0.048 | 0.0964 | No |
| 33 | COL11A2 | na | 12901 | 0.042 | 0.0838 | No |
| 34 | ITGA10 | na | 13121 | 0.039 | 0.0810 | No |
| 35 | SV2B | na | 13317 | 0.037 | 0.0787 | No |
| 36 | COMP | na | 14014 | 0.030 | 0.0614 | No |
| 37 | THBS1 | na | 14103 | 0.030 | 0.0615 | No |
| 38 | LAMB2 | na | 15383 | 0.018 | 0.0261 | No |
| 39 | GP1BA | na | 15439 | 0.017 | 0.0261 | No |
| 40 | ITGB4 | na | 15745 | 0.015 | 0.0186 | No |
| 41 | SDC4 | na | 16084 | 0.012 | 0.0100 | No |
| 42 | VTN | na | 16480 | 0.009 | -0.0006 | No |
| 43 | ITGA2B | na | 16498 | 0.009 | -0.0003 | No |
| 44 | LAMC3 | na | 16551 | 0.008 | -0.0011 | No |
| 45 | AGRN | na | 18607 | -0.009 | -0.0596 | No |
| 46 | SDC1 | na | 19019 | -0.013 | -0.0703 | No |
| 47 | ITGA8 | na | 19267 | -0.015 | -0.0760 | No |
| 48 | TNR | na | 20263 | -0.024 | -0.1026 | No |
| 49 | THBS3 | na | 20296 | -0.025 | -0.1013 | No |
| 50 | ITGA11 | na | 20534 | -0.027 | -0.1057 | No |
| 51 | IBSP | na | 21781 | -0.041 | -0.1380 | No |
| 52 | ITGA9 | na | 23027 | -0.055 | -0.1690 | No |
| 53 | LAMA4 | na | 23170 | -0.057 | -0.1679 | No |
| 54 | THBS2 | na | 23687 | -0.065 | -0.1770 | No |
| 55 | LAMA2 | na | 24087 | -0.071 | -0.1821 | No |
| 56 | TNN | na | 24439 | -0.076 | -0.1854 | No |
| 57 | COL2A1 | na | 25042 | -0.086 | -0.1951 | Yes |
| 58 | LAMA5 | na | 25084 | -0.087 | -0.1885 | Yes |
| 59 | TNXB | na | 25314 | -0.091 | -0.1869 | Yes |
| 60 | RELN | na | 25407 | -0.093 | -0.1813 | Yes |
| 61 | SV2A | na | 26010 | -0.104 | -0.1893 | Yes |
| 62 | HSPG2 | na | 26203 | -0.109 | -0.1851 | Yes |
| 63 | ITGA4 | na | 26362 | -0.112 | -0.1796 | Yes |
| 64 | GP1BB | na | 26487 | -0.114 | -0.1729 | Yes |
| 65 | COL4A1 | na | 26628 | -0.117 | -0.1664 | Yes |
| 66 | GP6 | na | 26640 | -0.118 | -0.1561 | Yes |
| 67 | COL6A3 | na | 26701 | -0.119 | -0.1471 | Yes |
| 68 | TNC | na | 26829 | -0.122 | -0.1399 | Yes |
| 69 | ITGB7 | na | 26886 | -0.123 | -0.1305 | Yes |
| 70 | COL6A1 | na | 27033 | -0.126 | -0.1233 | Yes |
| 71 | GP9 | na | 27159 | -0.129 | -0.1153 | Yes |
| 72 | ITGB6 | na | 27368 | -0.135 | -0.1092 | Yes |
| 73 | GP5 | na | 27798 | -0.145 | -0.1085 | Yes |
| 74 | VWF | na | 29046 | -0.180 | -0.1283 | Yes |
| 75 | THBS4 | na | 29503 | -0.195 | -0.1240 | Yes |
| 76 | SDC2 | na | 29806 | -0.204 | -0.1143 | Yes |
| 77 | COL6A6 | na | 30189 | -0.218 | -0.1058 | Yes |
| 78 | DAG1 | na | 30263 | -0.220 | -0.0881 | Yes |
| 79 | COL1A2 | na | 31565 | -0.272 | -0.1013 | Yes |
| 80 | SPP1 | na | 32451 | -0.323 | -0.0977 | Yes |
| 81 | LAMB4 | na | 32718 | -0.344 | -0.0745 | Yes |
| 82 | COL1A1 | na | 33804 | -0.457 | -0.0647 | Yes |
| 83 | COL3A1 | na | 33821 | -0.461 | -0.0237 | Yes |
| 84 | FN1 | na | 34246 | -0.555 | 0.0139 | Yes |
Table: GSEA details [plain text format]

  

Fig 2: KEGG\_ECM\_RECEPTOR\_INTERACTION      
 Blue-Pink O' Gram in the Space of the Analyzed GeneSet

  

Fig 3: KEGG\_ECM\_RECEPTOR\_INTERACTION: Random ES distribution      
 Gene set null distribution of ES for **KEGG\_ECM\_RECEPTOR\_INTERACTION**
